# The Performance of Spot Photoscreener in 6 to 10 Weeks Infants in China: A Cross-Sectional Study

**DOI:** 10.1155/2024/8817530

**Published:** 2024-05-11

**Authors:** Yaoling Li, Jing Li, Huiyu Wang, Mingyang Du, Lirong Wei, Teng Su, Gang Ding, Xuehan Qian, Ning Hua

**Affiliations:** ^1^Tianjin Key Laboratory of Retinal Functions and Diseases, Tianjin Branch of National Clinical Research Center for Ocular Disease, Eye Institute and School of Optometry, Tianjin Medical University Eye Hospital, Tianjin 300384, China; ^2^Beichen Women's and Children's Health Center, Tianjin 300384, China; ^3^Tianjin Binhai New Area Maternal and Child Health Care and Family Planning Service Center, Tianjin 300459, China

## Abstract

**Purpose:**

To compare the refractive errors measured by the Spot photoscreener (with or without cycloplegia) to cycloplegic retinoscopy in 6- to 10-week-old infants.

**Materials and Methods:**

101 right eyes from 101 healthy infants aged 6 to 10 weeks were recruited for this cross-sectional observational study. Refractive errors were measured using Spot photoscreener before and after cycloplegia, as well as cycloplegic retinoscopy. Comparisons between the refractive measurements were performed using one-way ANOVA with the post hoc Tukey HSD test or Kruskal–Wallis test with the Steel–Dwass test according to the data normality. Pearson's correlation test and 95% confidence intervals were calculated. The agreement was evaluated using a Bland–Altman plot with 95% limits of agreement of the differences.

**Results:**

Spot photoscreener was found to underestimate the spherical equivalent by 2.33 Diopters (D) in these infants. Following the induction of cycloplegia, the spherical equivalent measured by Spot photoscreener was in excellent agreement with cycloplegic retinoscopy with the mean difference of 0.01 D. Spot photoscreener overestimated cylindrical parameter by 0.2 D with poor agreement with cycloplegic retinoscopy no matter whether cycloplegia was induced. It had good agreement with cycloplegic retinoscopy in the *J*_0_ vector than the *J*_45_ vector measurement.

**Conclusions:**

With the induction of cycloplegia, Spot photoscreener can accurately evaluate spherical equivalent in hyperopic infants with mild-to-moderate astigmatism. While it may provide valuable measurements of astigmatism, discrepancies in cylinder and axis should be taken into account.

## 1. Introduction

Significant refractive errors, such as severe hyperopia and astigmatism, are commonly found in children with amblyopia, which is a sensory vision deficit with a prevalence of 2%–5% [1, 2]. Meanwhile, a variety of congenital ocular disorders co-occur with refractive errors, which may exacerbate visual disability if uncorrected [3–5]. Refractive error screening and reasonable correction are critical for amblyopia prevention and visual development in infants [6, 7]. Cycloplegic retinoscopy is considered the gold standard for pediatric refraction [8–11]. However, it requires clinicians with proficient skills and is usually time-consuming [12]. In addition, the poor cooperation of children makes it challenging to perform the retinoscopy and even problematic in infants.

Spot photoscreener (Welch Allyn, Skaneateles Falls, New York, USA) is a popular vision screening device for children. It uses an infrared camera to acquire images of the red reflex from binocular pupils simultaneously, from which the built-in software automatically calculates the refractive data. Due to its prompt data-reading and low cooperation requirement, it has been successfully performed in children younger than 3 years old [13]. Due to the disadvantages of cycloplegic retinoscopy in infants, we attempt to investigate whether Spot photoscreener could be used to assess refraction in young infants. In addition, to the best of our knowledge, there are few reports of comparison between Spot photoscreener and cycloplegic retinoscopy in infants. Thus, to explore the performance of Spot photoscreener in infants, our study compared the refractive errors obtained by Spot photoscreener with cycloplegia, Spot photoscreener without cycloplegia, and standard cycloplegic retinoscopy in healthy infants aged from 6 to 10 weeks.

## 2. Materials and Methods

This cross-sectional observational study was approved by the Institutional Ethics Board of Tianjin Medical University Eye Hospital (2019KY(L)-53) and followed the Declaration of Helsinki. All parents/guardians signed the written consent forms prior to the examinations being performed on participants.

Participants aged from 6 to 10 weeks were recruited from Beichen Women's and Children's Health Center (Tianjin, China) for a routine systemic health check-up between January 2020 and January 2021. Participants with systemic and ocular diseases, such as preterm, metabolic diseases, developmental retardance, cataracts, glaucoma, and ptosis, were excluded. Moreover, participants were also excluded if their spherical equivalent (SEQ) exceeded the range of −7.50 to +7.50 Diopters (D) or negative cylindrical value was over −3.00 D, which was out of the limit of Spot photoscreener measurement.

All participants underwent slit-lamp examination and Brückner test, followed by the binocular refractive reading with Spot photoscreener in a dimly lit room before cycloplegia. Compound tropicamide 0.5% and phenylephrine 0.5% (SINQI Pharmaceutical Co., Ltd., Shenyang, China) was used three times, with an interval of 5 minutes to induce cycloplegia until the pupil diameter reached 7-8 mm. Retinoscopy was performed 20 to 25 minutes following the final installation by a single proficient optometrist, using retinoscope (66 Vision Technology, Suzhou, Jiangsu Province, China) when the infants were held in the arms of their parents. To avoid potential bias, the optometrist was blinded to the results of the Spot photoscreener. After the cycloplegic refraction, a second Spot photoscreener measurement was performed.

All the measurements of retinoscopy and Spot photoscreener were repeated three times, and the average results were recorded for the final analysis. The parameters obtained included sphere (S), negative cylinder (C), and axis of the cylinder (A). Spherical equivalent (SEQ) and vector presentation of astigmatism *J*_0_ and *J*_45_ were calculated according to the following formulas: SEQ = S + C/2, *J*_0_  = (−C/2) *∗* cos (2 *∗* A), *J*_45_ = (−C/2) *∗* sin (2 *∗* A) [14]. According to SEQ value, emmetropia (≥−0.50 D to ≤0.50 D), hyperopia (>+0.50 D), or myopia (<−0.50 D) was defined [15].

### 2.1. Data Analysis and Statistics

All statistical analyses were performed with R software (version 4.3.3 (2024-02-29)). Comparisons between the measurements were performed using one-way ANOVA with the post hoc Tukey HSD test or Kruskal–Wallis test with the Steel–Dwass test according to the data normality. Agreement between cycloplegic retinoscopy and Spot photorefraction was assessed through Pearson's correlation test and the Bland–Altman plot. The 95% limit of agreement (LoA) was drawn according to the mean difference ± 1.96 SD [16]. The correlations were defined as weak if *r* was below 0.3, moderate if *r* was between 0.3 and 0.7, and strong if *r* was higher than 0.7. All statistical tests were two-tailed, and *P* values <0.05 were considered statistically significant. Differences of 1.00 D and 0.75 D or more for spherical and cylindrical measures between the three refraction methods were considered clinically significant [17–19].

## 3. Results

### 3.1. Participant Characteristics

A total of 133 infants were enrolled and 101 cases completed all the examinations. The reasons for withdrawal were unavoidable sleeping, crying of infants, and anxiety of parents. Among the 101 infants, 56 (55.4%) were females and 45 (44.6%) were males. The mean age was 7.9 ± 1.0 weeks, with ages ranged from 6 weeks to 10 weeks. All the right eyes of these infants were included and analyzed. 94 eyes (93.1%) were hyperopic, 5 eyes (5%) were emmetropic, and only 2 eyes (2%) were myopic.

### 3.2. Comparison of Refractive Errors between Spot Photoscreener and Cycloplegic Retinoscopy

Table 1 presents a comparative analysis of refractive errors among noncycloplegic Spot photoscreener, cycloplegic Spot photoscreener, and cycloplegic retinoscopy. The SEQ obtained from the noncycloplegic Spot photoscreener showed a statistically significant decrease compared to both the cycloplegic Spot photoscreener (*P* < 0.001) and cycloplegic retinoscopy (*P* < 0.001). In contrast, after cycloplegia, the SEQ measured by the Spot photoscreener did not significantly differ from that measured by retinoscopy (*P*=0.999).

For the cylindrical values, no statistically significant difference was observed among noncycloplegic Spot photoscreener, cycloplegic Spot photoscreener, and cycloplegic retinoscopy (*P*=0.099).

The comparison of *J*_0_ vector revealed no significant difference among noncycloplegic Spot photoscreener, cycloplegic Spot photoscreener, and cycloplegic retinoscopy (*P*=0.734). Similarly, for the *J*_45_ vector, no significant difference was observed among the noncycloplegic Spot photoscreener, cycloplegic Spot photoscreener, and cycloplegic retinoscopy (*P*=0.198).

### 3.3. Agreement between Spot Photoscreener and Cycloplegic Retinoscopy in the Detection of SEQ and Astigmatism

**Figure 1:**
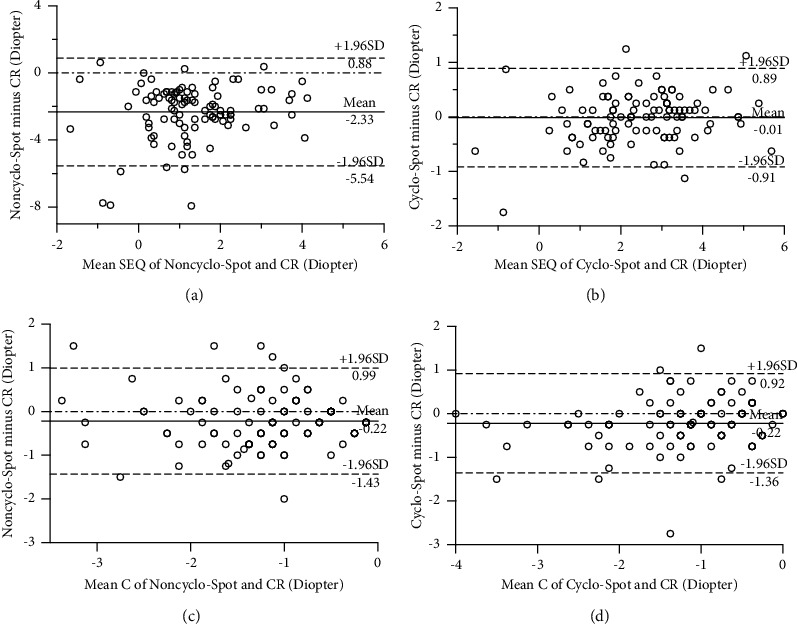
Bland–Altman plots of comparison of SEQ and cylinder between cycloplegic retinoscopy and Spot photoscreener with or without cycloplegia in 101 infants. (a and c) the Bland–Altman plots of comparison of SEQ and cylinder between noncycloplegic Spot photoscreener and cycloplegic retinoscopy; (b and d) the Bland–Altman plots of comparison of SEQ and cylinder between cycloplegic Spot photoscreener and cycloplegic retinoscopy. The middle line showed the mean difference, and the upper and lower lines represented 95% limits of agreement. SEQ, spherical equivalent. C, cylinder value; noncyclo-spot, noncycloplegic Spot photoscreener; cyclo-Spot, cycloplegic Spot photoscreener; CR, cycloplegic retinoscopy.

For SEQ, Pearson's correlation test (Table 2) revealed a weak correlation between noncycloplegic Spot photoscreener and cycloplegic retinoscopy (*r* = 0.330, *P*=0.001). The Bland–Altman plot (Figure 1(a)) showed that the mean difference of SEQ between the two methods was −2.33 D (95% LoA: −5.54 D to +0.88 D, *P* < 0.001), demonstrating poor agreement. However, with the induction of cycloplegia, Spot photoscreener exhibited a strong correlation with retinoscopy (*r* = 0.943, *P* < 0.001). The mean difference of SEQ was −0.01 D (95% LoA: −0.91 D to +0.89 D, *P*=0.999) according to the Bland–Altman plot (Figure 1(b)), indicating excellent good agreement in cycloplegic Spot photoscreener and retinoscopy.

For the measurement of cylindrical values, Pearson's correlation test demonstrated a moderate correlation between noncycloplegic Spot photoscreener and cycloplegic retinoscopy (*r* = 0.696, *P* < 0.001). After cycloplegia, Spot photoscreener displayed a strong correlation with retinoscopy (*r* = 0.780, *P* < 0.001). The Bland–Altman plots showed the mean difference of cylinder were −0.22 D (95% LoA: −1.43 D to +0.99 D, *P*=0.109) and −0.22 D (95% LoA: −1.36 D to +0.92 D, *P*=0.225) for noncycloplegic Spot photoscreener and cycloplegic Spot photoscreener compared to cycloplegic retinoscopy, respectively (Figures 1(c) and 1(d)), which demonstrated relatively low agreements with cycloplegic retinoscopy clinically.

In the analysis of *J*_0_, Pearson's correlation test indicated a strong correlation between Spot photoscreener and cycloplegic retinoscopy, irrespective of the induction of cycloplegia (*r* = 0.730, *P* < 0.001; *r* = 0.810, *P* < 0.001). However, for the *J*_45_, Pearson's correlation test demonstrated a moderate correlation between Spot photoscreener and cycloplegic retinoscopy (*r* = 0.451, *P* < 0.001; *r* = 0.644, *P* < 0.001), indicating that Spot photoscreener had a relatively weaker correlation with cycloplegic retinoscopy in *J*_45_ than in *J*_0_.

## 4. Discussion

In this study, we first compared the refractive errors measured by Spot photoscreener with those obtained by cycloplegic retinoscopy in very young infants aged from 6 to 10 weeks. The mean SEQ was found to be 2.49 ± 1.29 D in these infants, consistent with the reported data, indicating that most infants under 3 months of age exhibited approximately 2.00–3.00 D of hyperopia. [20–22] Our findings revealed that Spot photoscreener underestimated SEQ by 2.33 D compared to cycloplegic retinoscopy in these infants. With induction of cycloplegia by tropicamide 0.5% and phenylephrine 0.5% eye drops, the Spot photoscreener showed a good agreement with retinoscopy in SEQ, with the difference narrowing to −0.01 D. However, the Spot photoscreener exhibited a higher cylindrical power measurement with a difference of approximately 0.20 D, while it had a better ability to measure astigmatism in *J*_0_ than *J*_45_.

Various studies have reported that noncycloplegic Spot photoscreeners tend to underestimate hyperopia and overestimate myopia in children, with discrepancies in SEQ fluctuating from 0.30 D to over 4.50 D [23–27]. The variation in estimation has been attributed to factors, such as age, ethnicity, and the distribution of refractive errors. In our study, we found that the underestimation of the Spot photoscreener in Chinese infants under 3 months of age falls within the range reported in previous studies.

The accuracy of refractive evaluation relies on a stable accommodation, which cycloplegic eye drops can achieve. Spot photoscreener performs at about 1 meter of the working distance in front of children, and the focus on the colorful shining targets on the machine theoretically induces +1.00 D of accommodation. However, in practice, the accommodation in infants is much more variable than in adults [28–30], which may lead to unstable Spot photoscreener readings. Therefore, we selected short-term cycloplegic, tropicamide eye drops to block the ciliary muscle tone, which is safe with fewer side effects than atropine, and the drug manifests effective to suppress the accommodation in young infants [31].

With induction of cycloplegia, Spot photoscreener manifested a good correlation with retinoscopy and a good agreement with low bias in SEQ (0.01 D). Unlike the large gap of 95% LoA observed with the noncycloplegic Spot photoscreener (nearly over 6.00 D in SEQ), cycloplegic Spot photoscreener had a relatively narrow range of 95% LoA when compared to cycloplegic retinoscopy. This suggests that the performance of the cycloplegic Spot photoscreener is reliable for evaluating SEQ in young infants. Yakar [32] compared Spot photoscreener before and after induction of cycloplegia in 100 children aged 3–10 years. They found that the median SEQ increased from +0.25 D without cycloplegia to +1.00 D with cycloplegia (range: −3.25 D to +7.50 D), with the latter being comparable to the cycloplegic autorefractometer data (+1.00 D, range: −3.50 D to +7.38 D, ARK-1; Nidek, Tokyo, Japan). Additionally, sensitivity and negative predictive value for detecting significant refractive errors improved after cycloplegia induction.

However, Panda et al. [23] reported that the cycloplegic retinoscopy yielded results closer to the refraction obtained by the Spot photoscreener in undilated eyes (the difference in SEQ was −0.46 D for noncycloplegic Spot photoscreener while +0.55 D for the cycloplegic Spot photoscreener in 34 hyperopic children). Similar discrepancies were observed with other photorefractors under cycloplegia. After cycloplegia, Plusoptix A09 screener overestimated hyperopia by 0.39 D in 98 children under 6 years of age [33], and Plusoptix S04 screener overestimated by 1.46 D in 64 patients aged 2–19 years [34]. On the contrary, Schimitzek et al. [35] found that cycloplegia improved the accuracy of evaluating the SEQ with PowerRefII in 192 eyes from 104 patients (2–81 years) with the mean difference in SEQ was −0.12 ± 0.91 D. The discrepancies observed among these studies may be attributed to variations in age ranges and refractive errors. In our study, the included subjects were controlled in a narrow age range, which may explain the distinctive results compared to other studies.

In this study, the astigmatism was presented as cylinder, *J*_0_ and *J*_45_. *J*_0_ refers to the cylinder power set at orthogonally 90° and 180° meridians, representing Cartesian astigmatism, while *J*_45_ refers to a cross-cylinder set at 45° and 135°, representing oblique astigmatism [36]. We observed that the Spot photoscreener overestimated the cylindrical power by approximately 0.20 D, showing low agreement with cycloplegic retinoscopy. Furthermore, the Spot photoscreener had a good correlation with retinoscopy in *J*_0_ vector measurement but had a relatively weak correlation with measurement of retinoscopy in *J*_45_ vector, suggesting it had better capability to detect Cartesian astigmatism than oblique astigmatism in these young infants.

According to other studies, the Spot photoscreener detected higher cylindrical power by 0.16 D to 0.52 D without cycloplegia in children aged from 6 months to 18 years [26, 27, 37]. Kara and Petricli [38] observed slightly higher *J*_0_ values with the Spot photoscreener after cycloplegia in 305 children under 3 years of age, with a difference of less than 0.10 D compared to cycloplegic retinoscopy. They found a marked agreement in *J*_45_ vector results between Spot photoscreener and cycloplegic retinoscopy. Similarly, Panda et al. [23] found no difference in the axis of the cylinder between the cycloplegic Spot photoscreener and the cycloplegic retinoscopy, which is consistent with the report by Peterseim et al. [25].

In our study, we observed varying agreements between the Spot photoscreener and cycloplegic retinoscopy in cylinder, *J*_0_ and *J*_45_. The infrared camera of Spot photoscreener captures the binocular images of the red reflex, and the refractive data are automatically calculated by the built-in software according to the character of the red flex. However, young infants have very limited ability to concentrate and they occasionally failed to maintain focus on the camera. As a consequence, the camera may record the eccentric image of the red reflex rather than a centrally located one, leading to the misjudging of the refractive errors, particularly in astigmatism. Meanwhile, the infants were held in the arms of parents, and their chins might not be precisely aligned with the axis of the measurement, which could make it difficult to accurately assess the axis of astigmatism during retinoscopy. Although the optometrist in our study was skilled and well-experienced, personal bias of measurement could not be totally eliminated due to the limited ability of the young infants, especially in the judge of axis in these young infants.

The present study had a limited sample size, and most participants were healthy infants with mild hyperopia and mild-to-moderate astigmatism. Therefore, it represents the performance of Spot photoscreener in detecting this specific range of refractive error. Furthermore, only two myopic infants were included in the study, which means the comparison of cycloplegic Spot photoscreener with retinoscopy to measure myopia was nearly absent. Studies with a larger sample size consisting of a varying severity of refractive errors in infants are needed to further illustrate cycloplegic Spot photoscreener on refraction.

## 5. Conclusions

In summary, cycloplegic Spot photoscreener demonstrates a strong correlation and agreement with cycloplegic retinoscopy in spherical equivalent (SEQ) assessment in young infants with mild hyperopia and mild-to-moderate astigmatism. It can assist in measuring astigmatism, but the potential inaccuracies in cylinder and axis assessment need to be considered. Since the effectiveness of using the Spot photorefraction after cycloplegia is still debated, conducting research with bigger sample sizes might provide clarity on the impact of cycloplegic Spot photoscreener on children's refraction.

## Figures and Tables

**Table 1:** Comparison of spherical equivalent and astigmatism among noncycloplegic Spot photoscreener, cycloplegic Spot photoscreener, and cycloplegic retinoscopy.

	Noncyclo-Spot			Cyclo-Spot			CR			∆	Noncyclo-Spot vs. CR	Cyclo-Spot vs. CR	Noncyclo-Spot vs. cyclo-Spot
	Mean (SD)	Min	Max	Mean (SD)	Min	Max	Mean (SD)	Min	Max	*P* value	*P* value	*P* value	*P* value
SEQ (D)	0.16 (1.51)	−4.75	3.75	+2.48 (1.39)	−1.88	5.63	+2.49 (1.30)	−1.25	6.00	<0.001	<0.001	0.999	<0.001
C (D)	−1.33 (0.78)	−3.50	−0.25	−1.33 (0.92)	−4.25	0.00	−1.11 (0.81)	−4.00	0.00	0.099	0.109	0.225	0.927
*J*_0_ (D)	+0.48 (0.50)	−1.00	1.75	+0.53 (0.52)	−0.62	2.12	+0.45 (0.48)	−0.75	1.97	0.734	0.858	0.722	0.974
*J*_45_ (D)	+0.09 (0.32)	−0.54	1.29	+0.02 (0.32)	−0.84	0.86	−0.01 (0.20)	−0.62	0.65	0.198	0.173	0.757	0.517

Noncyclo-spot, noncycloplegic spot photoscreener; cyclo-Spot, cycloplegic Spot photoscreener; CR, cycloplegic retinoscopy; SEQ, spherical equivalent; C, cylinder value; *J*_0_, Jackson cross-cylinder, axis at 90°and 180°; *J*_45_, Jackson cross-cylinder, axis at 45° and 135°; vs., versus; SD, standard deviation; Min, minimum; Max, maximum; D, diopter; ∆, comparison among noncycloplegic Spot photoscreener, cycloplegic Spot photoscreener, and cycloplegic retinoscopy.

**Table 2:** Correlation between cycloplegic retinoscopy and Spot photoscreener with or without cycloplegia.

		Pearson's *r*	95% CI		*P* value
			Lower	Upper	
SEQ	Noncyclo-Spot-CR	0.330	0.128	0.534	0.001
	Cyclo-Spot-CR	0.943	0.920	0.962	<0.001

C	Noncyclo-Spot-CR	0.696	0.563	0.799	<0.001
	Cyclo-Spot-CR	0.780	0.633	0.868	<0.001

*J*_0_	Noncyclo-Spot-CR	0.730	0.597	0.824	<0.001
	Cyclo-Spot-CR	0.810	0.699	0.882	<0.001

*J*_45_	Noncyclo-Spot-CR	0.451	0.205	0.638	<0.001
	Cyclo-Spot-CR	0.644	0.499	0.766	<0.001

SEQ, spherical equivalent; C, cylindrical value; *J*_0_, Jackson cross-cylinder, axis at 90° and 180°; *J*_45_, Jackson cross-cylinder, axis at 45° and 135°; noncyclo-spot, noncycloplegic Spot photoscreener; Cyclo-Spot, cycloplegic Spot photoscreener; CR, cycloplegic retinoscopy; CI, confidence interval.

## Data Availability

The data used to support the findings of this study are available from the corresponding author upon request.
